# Baloxavir-Based Combination Therapy for Protection against Influenza A(H5N1) Clade 2.3.4.4b Virus Infection in Mice

**DOI:** 10.3201/eid3210.260186

**Published:** 2026-10

**Authors:** Seong Cheol Min, Ju Ryeong Lee, Beom Kyu Kim, Ji-Hyun Park, Dong Gyu Lee, Gi Chan Lee, Se Hee An, Santosh Chokkakula, Aman Jain, Young Ki Choi, Yun Hee Baek, Min-Suk Song

**Affiliations:** Chungbuk National University College of Medicine, Chungbuk, South Korea (S.C. Min, J.R. Lee, B.K. Kim, J.-H. Park, D.G. Lee, G.C. Lee, S.H. An, S. Chokkakula, A. Jain, Y.H. Baek, M.-S. Song); Korea Virus Research Institute Yuseong-gu, Daejeon, South Korea (Y.K. Choi)

**Keywords:** Influenza, viruses, zoonoses, respiratory infections, influenza A virus, H5N1 subtype, baloxavir, oseltamivir, molnupiravir, drug combinations

## Abstract

Highly pathogenic avian influenza A(H5N1) clade 2.3.4.4b virus continues to cause animal outbreaks and sporadic zoonotic infections. In a mouse model of lethal influenza disease, we compared oseltamivir, baloxavir, and molnupiravir monotherapies with 2-drug combinations. Baloxavir-based combinations improved survival, reduced lung viral loads, and prevented extrapulmonary dissemination, supporting H5N1 preparedness strategies.

Highly pathogenic avian influenza A(H5N1) clade 2.3.4.4b virus continues to cause widespread outbreaks and sporadic zoonotic infections, underscoring the need to optimize antiviral strategies (*1*–*3*). Licensed influenza antiviral drugs can reduce disease severity but might be compromised in severe infections by high viral burdens, treatment delays, and treatment-emergent resistance, motivating evaluation of combination regimens (*4*–*7*). Recent H5N1 treatment studies using mouse models suggest antiviral performance can vary by exposure route and disease progression (*8*–*11*). To inform preparedness-oriented selection, we compared direct-acting antiviral drugs from distinct classes in a lethal mouse model. We tested monotherapies and 2-drug combinations of 2 licensed influenza antiviral agents, oseltamivir phosphate (OSP; neuraminidase inhibitor) and baloxavir acid (BXA; cap-dependent endonuclease inhibitor), and molnupiravir (MPV; nucleoside analog) to assess whether combinations provided synergistic benefit.

## The Study

We challenged 6- to 8-week-old female BALB/c mice intranasally with A/mink/Spain/3691–8_22VIR10586–10/2022 (5 LD_50_ [50% median lethal dose]) in a 50-µL inoculum (90 TCID_50_ [50% tissue culture infectious dose]) (Appendix). We treated mice at 6 hours postinfection (hpi). We administered OSP and MPV orally twice daily for 5 days and administered BXA once subcutaneously. For the low-dose comparison, mice received OSP 25 mg/kg, MPV 25 mg/kg, or BXA 20 mg/kg, and corresponding 2-drug pairs at the same dosages: OSP/MPV, BXA/OSP, BXA/MPV. We monitored body weight and survival through 18 days postinfection (dpi) (n = 7) and quantified infectious virus titers in lung, brain, and heart by TCID_50_ assay at 4 and 6 dpi (n = 3/time point) (Figure 1; Appendix Tables 1–5).

**Figure 1:**
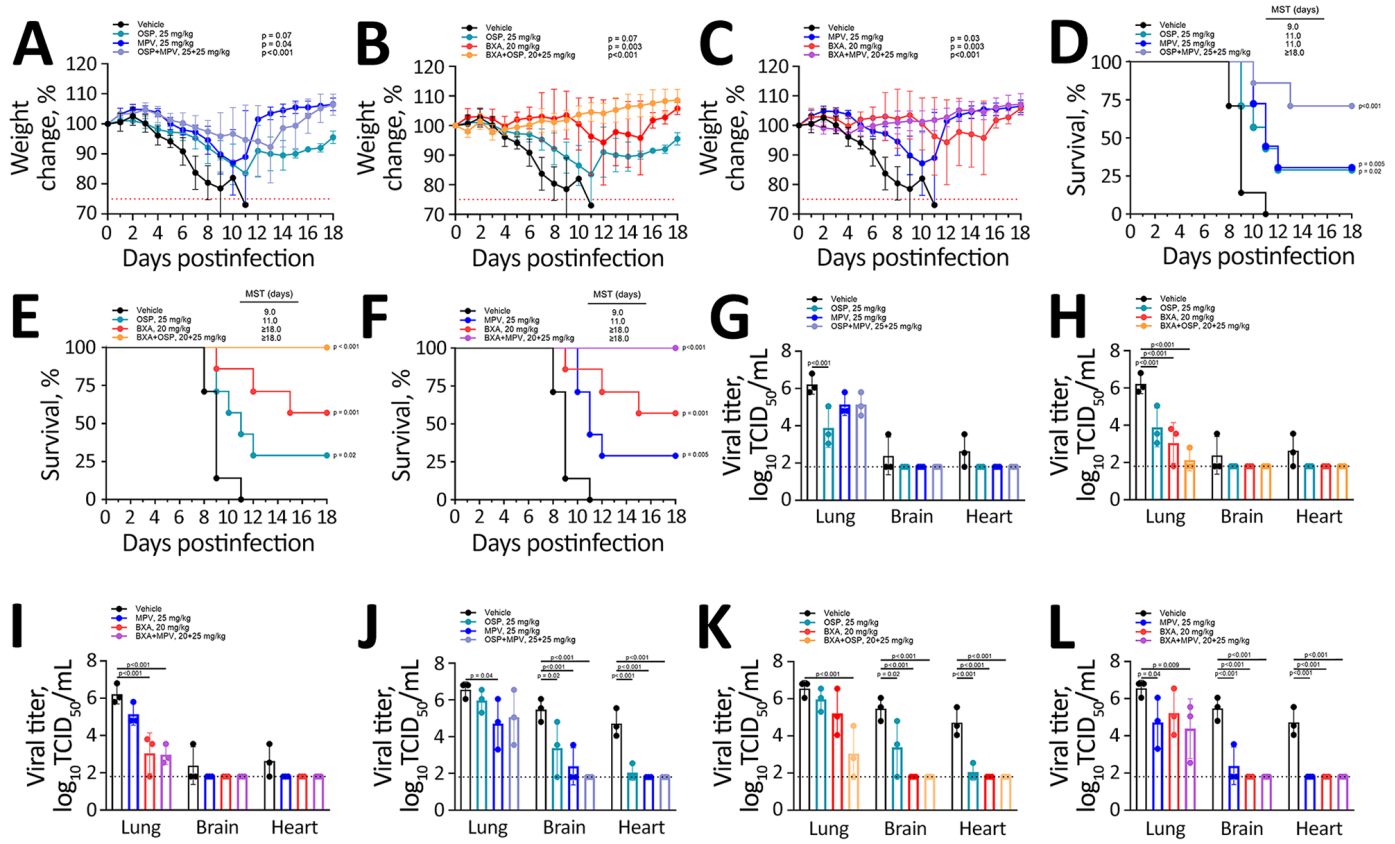
Therapeutic efficacy of baloxavir-based combinations and monotherapies for treating influenza A(H5N1) clade 2.3.4.4b virus infection in mice. Drug names and doses are provided in the keys (e.g., BXA+MPV 20+25 mg/kg indicates BXA/MPV at a dose of 20/25 mg/kg). A–C) Body weight change (mean +SD; n = 7/group) through 18 days postinfection: vehicle, MPV 25 mg/kg, OSP 25 mg/kg, and OSP/MPV 25/25 mg/kg (A); vehicle, OSP 25 mg/kg, BXA 20 mg/kg, and BXA/OSP 20/25 mg/kg (B); vehicle, MPV 25 mg/kg, BXA 20 mg/kg, and BXA/MPV 20/25 mg/kg (C). Symbols represent group means; error bars indicate SDs. Red dotted horizontal line indicates the humane endpoint threshold. D–F) Kaplan–Meier survival (n = 7/group) corresponding to panels A–C; MST values shown in each panel. G–I) Viral titers in lung, brain, and heart at 4 days postinfection (n = 3/group). J–L) Viral titers at 6 dpi (n = 3/group). Bars show group means with individual animals overlaid; dashed line denotes assay limit of detection (1.801 log_10_ TCID_50_/mL). BXA, baloxavir acid; MPV, molnupiravir; MST, median survival time; OSP, oseltamivir phosphate; SD, standard deviation; TCID_50_, 50% tissue culture infectious dose.

Vehicle-treated mice exhibited rapid weight loss and uniform death. In monotherapy low-dose treatments, OSP or MPV conferred partial protection (2/7 survival each), whereas BXA administered as a single dose was more protective (4/7 survival) and more consistently limited weight loss (Figure 1, panels A–F). Dose-escalation to 50 mg/kg monotherapies also improved outcomes but did not provide 100% protection (Appendix Figures 2, 3), consistent with prior reports showing therapeutic benefit of antiviral drugs in H5N1 mouse infection models (*8*–*11*). Accordingly, we evaluated 2-drug regimens in which BXA was maintained at 20 mg/kg and partnered with OSP or MPV at either 25 or 50 mg/kg. Across all three 2-drug regimens—OSP/MPV (25/25 mg/kg and 50/50 mg/kg), BXA/OSP (20/25 mg/kg and 20/50 mg/kg), and BXA/MPV (20/25 mg/kg and 20/50 mg/kg)—combination treatment stabilized body weight and markedly improved survival relative to the corresponding monotherapies (Figure 1, panels A–F; Appendix Figure 3). BXA-containing combinations showed the greatest protection, and no deaths (7/7 survival) occurred through 18 dpi at both dose pairs, whereas OSP/MPV did not show complete protection (Figure 1, panels A–F; Appendix Figure 3). Bliss-independence analysis (*12*) of end-of-study survival (18 dpi; n = 7/group) showed positive ΔBliss estimates for BXA-containing combinations, most clearly at the 20/25 mg/kg dose pairs. At the 20/50 mg/kg dose pairs, ΔBliss values remained positive but were smaller, consistent with an attenuated interaction at higher antiviral exposure. OSP/MPV showed a weaker, dose-dependent interaction (Appendix Table 6).

We observed that clinical benefit paralleled tissue-level virologic control and prevention of extrapulmonary dissemination (Figure 1, panels G–L). At 4 dpi, monotherapy with BXA and OSP, but not MPV, reduced lung titers; brain and heart titers were near or below the limit of detection (LOD), consistent with limited dissemination at that timepoint. By 6 dpi, vehicle-treated mice showed high lung viral loads accompanied by detectable virus in brain and heart. In treated animals, BXA provided the most consistent tissue-level control, keeping viral titers in brain and heart at the LOD; 25 mg/kg OSP or MPV permitted occasional instances of replication in the brain.

Combination therapy further inhibited residual dissemination. At 4 dpi, lung titers in combination groups frequently approached the LOD, most prominently for BXA-containing pairs, whereas brain and heart remained at or near the LOD. By 6 dpi, low lung titers were sustained, and systemic spread was largely abolished in BXA-containing groups. In contrast, OSP/MPV combinations showed comparatively weaker lung viral clearance and survival benefit. Nevertheless, those combinations converted partial clinical protection into near-complete or complete survival, reduced pulmonary titers, and prevented neuroinvasion and detectable heart virus that were still observed under some monotherapy conditions.

We conducted histopathology and immunohistochemistry and transcriptomic profiling of lung and brain at 6 dpi (n = 3/group) (Figures 2–4). In representative sections, vehicle-treated mice showed prominent influenza nucleoprotein immunoreactivity in both lung and brain (Figure 2), consistent with extensive tissue antigen burden during lethal infection (Figure 1). Across monotherapy groups, nucleoprotein staining and associated tissue involvement appeared reduced but remained detectable. However, combination therapy showed markedly diminished to near-absent nucleoprotein signal in both organs, most consistently the BXA-containing pairs, supporting a tissue-level correlate for the observed improved survival and restricted dissemination. Concordantly, RNA sequencing at 6 dpi demonstrated treatment-associated clustering of transcriptomic profiles by principal component analysis, showing separation between vehicle-treated and antiviral-treated groups. BXA-containing combinations showed tighter clustering than monotherapies in both lung and brain, consistent with a more uniform host transcriptional response under the most protective regimens (Figure 3, panel A; Figure 4, panel A). Pathway analysis identified differential enrichment of host innate immune, inflammatory, and neuronal/synaptic pathways within both tissues. Combination therapies, particularly BXA-containing regimens, showed reduced enrichment of pathways relevant to type II interferon, innate immune response, and inflammatory response compared with monotherapies. Of note, those combinations showed relative preservation of neuronal and synaptic pathways, such as cognition, memory, and vesicle-mediated transport, and profiles shifted closer to uninfected controls (Figure 3, panel B; Figure 4, panel B). 

**Figure 2:**
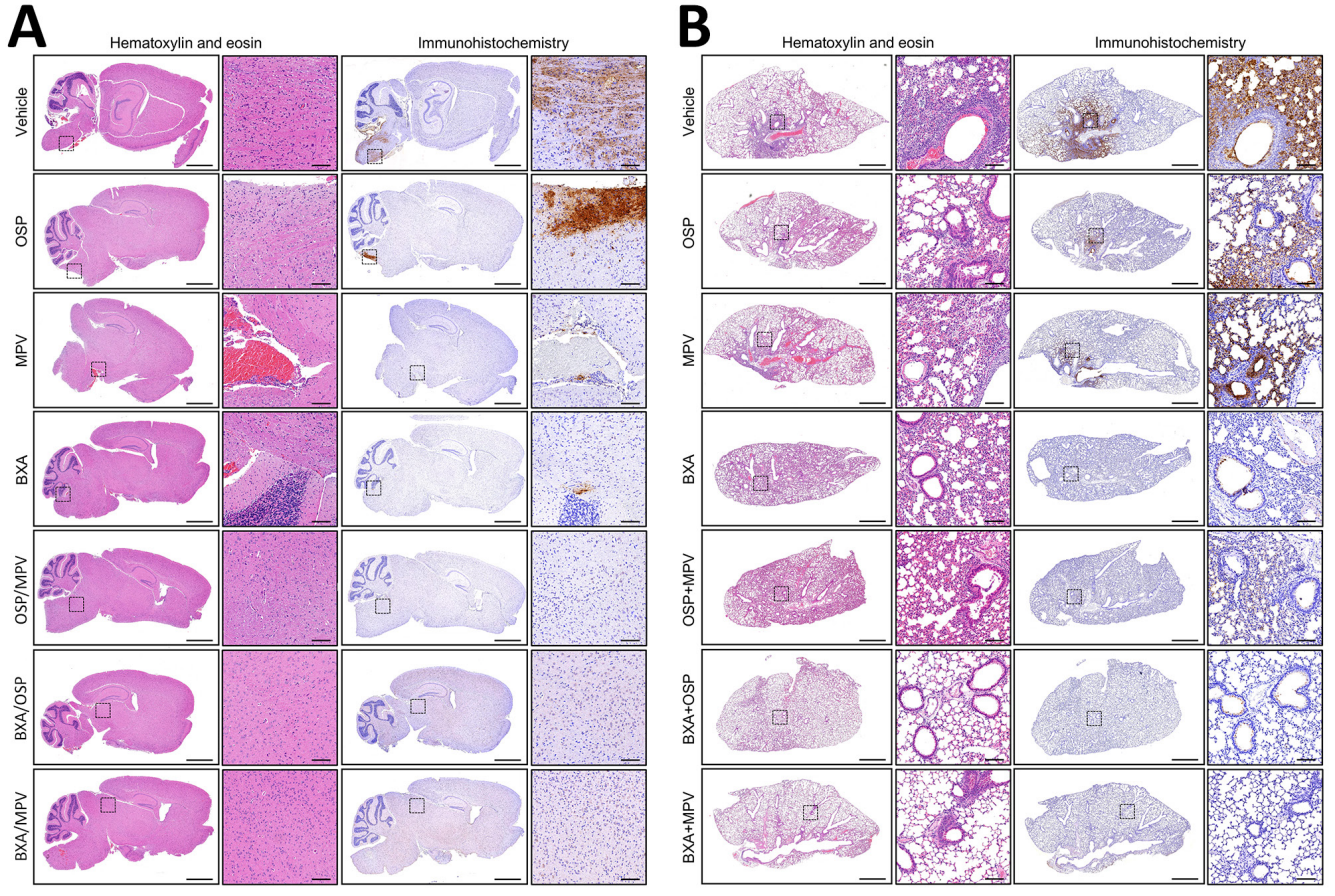
Lung and brain histopathology and influenza A nucleoprotein immunoreactivity at 6 dpi after monotherapy or combination therapy in a study of treating influenza A(H5N1) clade 2.3.4.4b virus infection in mice. A) Brain sections; B) lung sections. Sections from vehicle, OSP, MPV, BXA, OSP/MPV, BXA/OSP, and BXA/MPV groups. For each group, representative hematoxylin and eosin images (left) and immunohistochemistry for influenza A nucleoprotein (right; 3,3′-diaminobenzidine with hematoxylin counterstain) are shown as whole-section views with corresponding higher-magnification insets (boxed regions). Scale bars indicate 1.25 mm for whole-section views; and 100 μm for higher-magnification insets. BXA, baloxavir acid; MPV, molnupiravir; OSP, oseltamivir phosphate.

**Figure 4:**
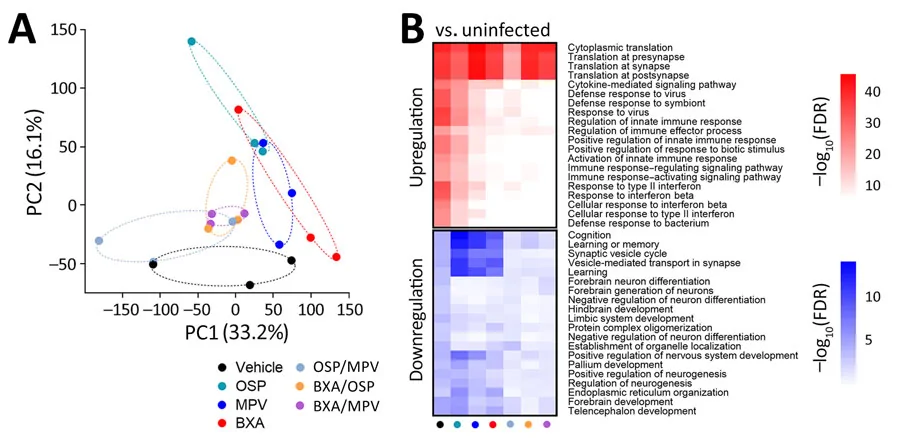
Brain transcriptomic responses in comparison of baloxavir-based combinations and monotherapies for treating influenza A(H5N1) clade 2.3.4.4b virus infection in mice. Response at 6 days postinfection show treatment-associated separation and attenuation of infection-associated pathway perturbations under combination therapy. RNA sequencing performed on brain harvested at 6 days postinfection from same experimental groups as Figure 1 (n = 3/group). A) Principal component analysis of transcriptomic profiles for brain; each point represents an individual mouse, and ellipses denote within-group dispersion. Percent variance explained by PC1 and PC2 indicated on axes. B) Pathway enrichment results comparing each infected treatment group versus uninfected controls for brain, displayed as heatmaps for representative upregulated and downregulated biologic pathways. Color intensity represents −log_10_ (false discovery rate) for enriched terms. BXA, baloxavir acid; FDR, false discovery rate; MPV, molnupiravir; OSP, oseltamivir phosphate.

**Figure 3:**
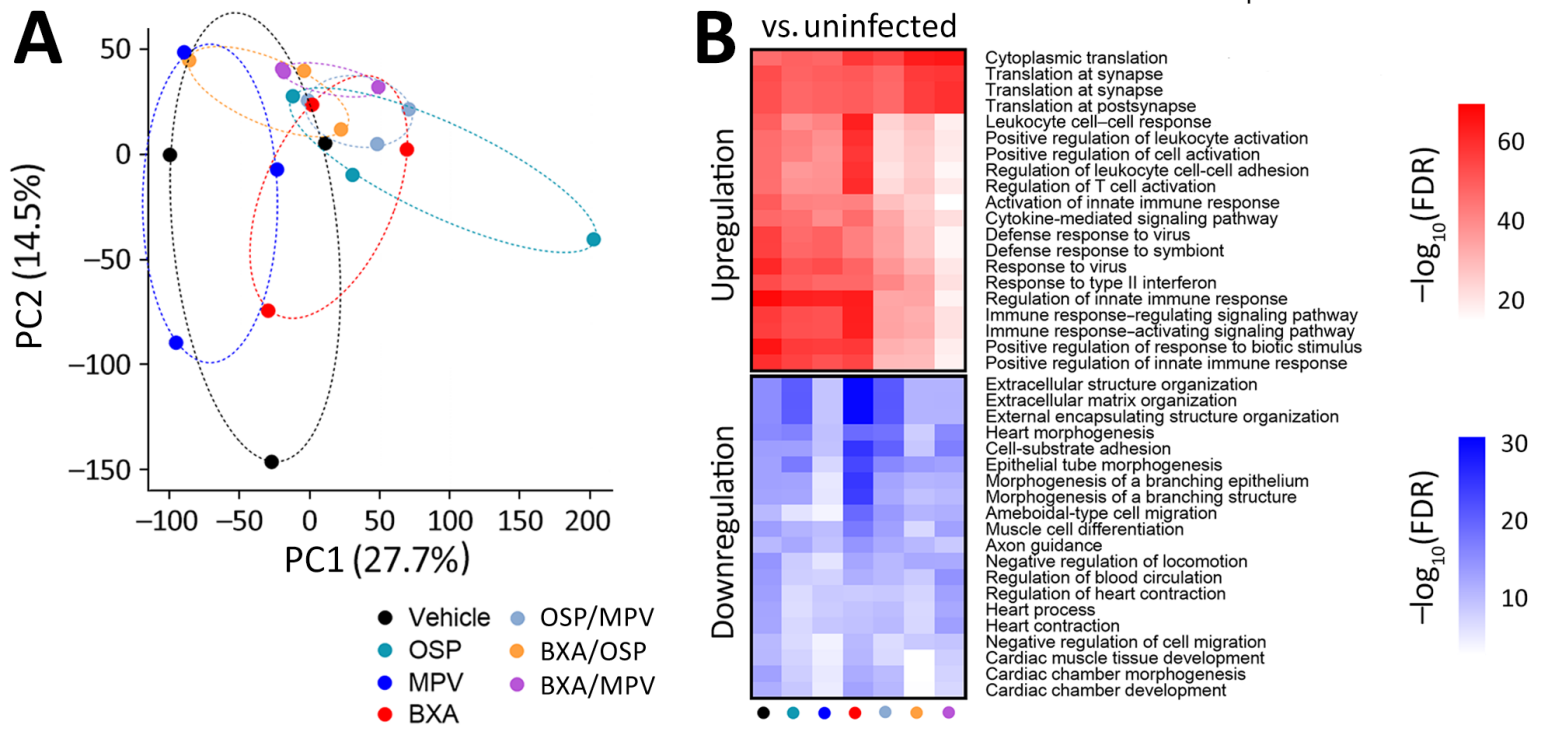
Lung transcriptomic responses at 6 days postinfection in comparison of baloxavir-based combinations and monotherapies for treating influenza A(H5N1) clade 2.3.4.4b virus infection in mice. Responses show treatment-associated separation and attenuation of infection-associated pathway perturbations under combination therapy. RNA sequencing performed on lung harvested at 6 days postinfection from the same experimental groups as Figure 1 (n = 3/group). A) Principal component analysis of transcriptomic profiles for lung; each point represents an individual mouse, and ellipses denote within-group dispersion. Percent variance explained by PC1 and PC2 indicated on axes. B) Pathway enrichment results comparing each infected treatment group versus uninfected controls for lung, displayed as heatmaps for representative upregulated and downregulated biologic pathways. Color intensity represents −log_10_ (false discovery rate) for enriched terms. BXA, baloxavir acid; FDR, false discovery rate; MPV, molnupiravir; OSP, oseltamivir phosphate.

Limitations of our investigation included early treatment initiation (6 hpi), modest sample sizes for tissue titration, and the use of MPV, which is not licensed for influenza (*13*). Exploratory single nucleotide polymorphism analysis of 6-dpi lung viral RNA did not identify canonical PA-I38 or NA-H275Y substitutions in antiviral-treated groups; however, we did not perform targeted deep sequencing and phenotypic susceptibility testing. Because we did not evaluate delayed treatment at 24 or 48 hpi, further studies are needed to determine whether BXA-based combinations retain efficacy when initiated later after infection. Nonetheless, the consistency of BXA-containing combination benefit across dosing regimens (Figure 1; Appendix Figure 3) and across endpoints (clinical course, survival, and tissue infectious virus) supports further evaluation in settings closer to clinical use, including delayed initiation, additional clade 2.3.4.4b isolates, and transmission-relevant models.

## Conclusions

In a lethal clade 2.3.4.4b H5N1 mouse model, monotherapies with OSP, MPV, or BXA improved outcomes in a dose-dependent manner but did not consistently prevent death or extrapulmonary spread. In contrast, 2-drug combinations, particularly those containing BXA, achieved complete survival at both dosing regimens and suppressed pulmonary replication to near LOD while preventing neuroinvasion and cardiac dissemination. Those findings are consistent with recent clade 2.3.4.4b A(H5N1) studies showing strong in vivo activity of BXA and BXA-containing regimens and extend those observations by directly comparing BXA/OSP and BXA/MPV with OSP/MPV in a lethal mouse model (*8*,*10*,*11*,*14*). Our findings extend recent antiviral-combination studies in less virulent influenza models by showing that BXA-containing combinations provided the strongest protection in a lethal clade 2.3.4.4b H5N1 model (*14*,*15*). Together, our findings provide an experimentally grounded rationale to prioritize BXA-based 2-drug regimens as a preparedness-oriented option for emergent H5N1 infections when antiviral treatment is initiated early after infection, particularly where severe disease or resistance risk can compromise single-agent performance. 

Appendix Additional information for comparison of baloxavir-based combinations and monotherapies for treating influenza A(H5N1) clade 2.3.4.4b virus infection in mice.
