# Supplementary material for: Baloxavir-Based Combination Therapy for Protection against Influenza A(H5N1) Clade 2.3.4.4b Virus Infection in Mice
Source: Emerg Infect Dis. 2026 Oct;32(10):1730–5. doi: 10.3201/eid3210.260186 (PMC13632834; doi:10.3201/eid3210.260186)
Supplement: Supplementary file 1 [file 26-0186-Techapp-s1.pdf]

Article DOI: http://doi.org/10.3201/eid3210.260186

EID cannot ensure accessibility for supplementary materials supplied by authors. Readers who have difficulty accessing supplementary content should contact the authors for assistance.

Baloxavir-Based Combination Therapy for Protection against Influenza A(H5N1) Clade 2.3.4.4b Virus Infection in Mice

Appendix

Methods

Ethics statement

All animal experiments were approved by the Institutional Animal Care and Use Committee of Chungbuk National University (CBNUA-24–0059–01) and conducted in accordance with institutional guidelines. Experiments involving highly pathogenic avian influenza virus (HPAI) clade 2.3.4.4b were performed in an approved biosafety level 3-plus (BSL-3+) facility at Chungbuk National University, following regulations of the Ministry of Food and Drug Safety and the Korea Disease Control and Prevention Agency, Republic of Korea.

Cells and virus

Madin-Darby canine kidney (MDCK) cells (ATCC, CCL-34) were maintained in minimal essential medium (MEM; Corning) supplemented with 10% fetal bovine serum (FBS; Atlas), 1% antibiotic–antimycotic solution (GIBCO), and 1% MEM vitamin solution (GIBCO) at 37°C with 5% CO_2. The A/mink/Spain/3691–8_22VIR10586–10/2022 (H5N1 clade 2.3.4.4b) virus was generated using a reverse-genetics system as previously described (*1*). The reverse-genetics virus was generated using sequences derived from A/mink/Spain/3691–8_22VIR10586–10/2022; the 8 gene segments are available in GISAID under accession numbers EPI2220590–EPI2220597. Briefly, HEK293T and MDCK cells were co-transfected with eight bidirectional plasmids (500 ng each) and incubated in Opti-MEM containing TPCK-treated trypsin at 37°C for 48–72 h. Virus-containing supernatants

were propagated in 10-day-old specific-pathogen-free embryonated chicken eggs at 35°C for 36–48 h. Allantoic fluids were clarified by centrifugation and stored at −80°C. Viral titers were determined by 50% tissue culture infectious dose ($TCID_{50}$) assay on MDCK cells.

Antiviral compounds

Oseltamivir phosphate (OSP), molnupiravir (MPV), and baloxavir acid (BXA) were purchased from MedChemExpress (USA). OSP and MPV were dissolved in Dulbecco's phosphate-buffered saline (DPBS), and BXA was dissolved in 0.5% (w/v) methylcellulose. All stock solutions were stored at −20°C and freshly diluted before use.

Animals and infection

Six- to 8-week-old female BALB/c mice (Samtako, Republic of Korea) were used. The 50% mouse lethal dose (MLD_{50}) was determined by intranasal infection with 10-fold serial dilutions of virus (Appendix Figure 1). For therapeutic studies, mice were anesthetized with isoflurane and intranasally inoculated with 5 MLD_{50} (90 $TCID_{50}$ in 50 μL) of virus.

Bodyweight and survival were monitored daily up to 18 days post-infection (dpi). Mice reaching humane endpoints were euthanized. Subsets of animals were euthanized at 4 dpi and 6 dpi for tissue collection.

Antiviral treatment regimens

Therapeutic administration was initiated at 6 hours post-infection (hpi) and delivered orally or subcutaneously in a consistent volume of 100 μL per mouse. Oseltamivir phosphate (OSP) and molnupiravir (MPV) were administered by oral gavage twice daily for 5 consecutive days (every 12 hours; total daily doses of 50 or 100 mg/kg/day for the 25 or 50 mg/kg per-dose regimens, respectively), whereas baloxavir acid (BXA) was administered as a single subcutaneous injection at 6 hpi (single total dose of 20 or 50 mg/kg; no repeated dosing). For monotherapy experiments, mice received OSP or MPV at 25 or 50 mg/kg per dose (50 or 100 mg/kg/day, every 12 hours; BID × 5 days) or BXA at 20 or 50 mg/kg (single total dose). In this study, the low-dose comparison refers to OSP or MPV at 25 mg/kg per dose and BXA at 20 mg/kg, whereas the high-dose comparison refers to OSP or MPV at 50 mg/kg per dose; BXA was maintained at 20 mg/kg in BXA-containing combination regimens. For combination therapy, two-drug regimens were evaluated using fixed dose pairs, with each drug administered sequentially in a volume of 100 μL per mouse (for a total of 200 μL per mouse). OSP+MPV was given at 25+25 mg/kg or 50+50 mg/kg per dose (total daily doses of 50+50 or 100+100 mg/kg/day, respectively; every 12 hours for 5 days). For

BXA-containing combinations, BXA was maintained at 20 mg/kg (single total dose) and co-administered with either OSP or MPV at 25 or 50 mg/kg per dose (50 or 100 mg/kg/day, every 12 hours for 5 days), yielding BXA+OSP and BXA+MPV dose pairs of 20+25 and 20+50 mg/kg. These dose levels were selected to enable within-study comparisons among monotherapies and 2-drug combinations, with OSP and MPV tested at 25 or 50 mg/kg per dose and BXA administered as a single subcutaneous dose maintained at 20 mg/kg in BXA-containing combination regimens. The subcutaneous BXA route and dose were selected based on prior murine PK/PD data showing favorable BXA exposure after subcutaneous administration and on recent clade 2.3.4.4b A(H5N1) mouse studies that used subcutaneous BXA across overlapping dose ranges (*2–4*). These regimens were not intended to represent clinically equivalent human doses in the absence of compound-specific pharmacokinetic/pharmacodynamic bridging. For each dosing regimen, clinical outcomes (bodyweight and survival) were monitored through 18 days post-infection (n = 7/group), and separate cohorts were euthanized at 4 and 6 dpi for tissue virology and ancillary analyses (n = 3/group/time point).

Viral titration

Lung, brain, and heart tissues were homogenized in MEM and clarified by centrifugation. Viral titers were quantified by $TCID_{50}$ assay on MDCK cells and expressed as $\log_{10}$ $TCID_{50}$/mL. The assay limit of detection was defined as the lowest dilution yielding cytopathic effect.

Synergistic effect analysis (Bliss independence)

To quantify interaction between antivirals in combination therapy, Bliss independence was applied using end-of-study survival as the efficacy endpoint. For each treatment, efficacy (E) was defined as the proportion of surviving animals at 18 dpi. The expected additive effect under Bliss independence was calculated as:

$$E_{exp} = E_A+E_B-(E_A\times E_B)$$

where E_A and E_B denote efficacies of the respective monotherapies. The observed combination effect (E_{obs}) was compared with E_{exp}, and the interaction term was expressed as:

$$\Delta Bliss = E_{obs} - E_{exp}$$

Positive ΔBliss values indicate effects above Bliss-expected additivity, values near zero indicate additivity, and negative values indicate antagonism. To estimate uncertainty,

bootstrap resampling (10,000 iterations) was performed on survival outcomes to derive 95% confidence intervals for ΔBliss. Detailed ΔBliss estimates, expected Bliss effects, and bootstrap confidence intervals are provided in Appendix Table 6.

Histopathology and immunohistochemistry

Tissues were fixed in 10% neutral-buffered formalin, paraffin-embedded, and sectioned at 3 μm. For histology, sections were stained with hematoxylin and eosin (H&E). For immunohistochemistry (IHC), antigen retrieval was performed in EDTA buffer (pH 9.0) at 100°C for 20 min. Endogenous peroxidase was quenched, and sections were incubated with rabbit polyclonal anti-influenza A nucleoprotein (NP) antibody (Invitrogen PA5–32242; 1:750), followed by HRP-labeled anti-rabbit polymer (Agilent Dako). Signals were developed using DAB and counterstained with Mayer's hematoxylin. Whole-slide images were acquired using a KF-PRO-020 scanner (KFBIO).

RNA sequencing

Total RNA was isolated from lung and brain tissues at 6 dpi using the RNeasy Mini Kit (Qiagen). Library preparation and sequencing were performed by JSLink Inc. (Seoul, Republic of Korea) using the NovaSeq 6000 platform (paired-end 2 × 100 bp). Reads with ≥80% bases at Q30 were retained. Pseudoalignment was performed against the GRCm38 reference using Kallisto (v0.46.1) (*5*). Genes with counts per million (CPM) ≥2 in at least one replicate were retained, and expression values were normalized using the trimmed mean of M-values (TMM) method in edgeR (*6*,*7*).

Differential expression and pathway analysis

Differentially expressed genes (DEGs) were identified using edgeR with a generalized linear model. DEGs were defined by $|\log_2$ fold change$| \geq 1$ and false discovery rate (FDR) <0.05 (Benjamini–Hochberg correction). Functional enrichment was assessed using DAVID (*8*) and clusterProfiler for Gene Ontology (BP, MF, CC) and KEGG pathways. Enrichment significance was set at $p < 0.05$. Viral SNPs in lung RNA-seq reads collected at 6 dpi were screened exploratorily for known reduced-susceptibility markers, focusing on PA substitutions associated with reduced BXA susceptibility and NA substitutions associated with reduced OSP susceptibility.

Statistical analysis

All statistical analyses were performed using GraphPad Prism 10. Survival curves were analyzed using the Kaplan–Meier method with log-rank (Mantel–Cox) tests.

Bodyweight trajectories were summarized by area under the curve (AUC) and compared using one-way ANOVA with Dunnett's multiple-comparisons test versus vehicle. Viral titers were analyzed by two-way ANOVA with Dunnett's multiple-comparisons test. $p < 0.05$ was considered statistically significant.

References

1. Hoffmann E, Neumann G, Kawaoka Y, Hobom G, Webster RG. A DNA transfection system for generation of influenza A virus from eight plasmids. Proc Natl Acad Sci U S A. 2000;97:6108–13. PubMed https://doi.org/10.1073/pnas.100133697

2. Ando Y, Noshi T, Sato K, Ishibashi T, Yoshida Y, Hasegawa T, et al. Pharmacokinetic and pharmacodynamic analysis of baloxavir marboxil, a novel cap-dependent endonuclease inhibitor, in a murine model of influenza virus infection. J Antimicrob Chemother. 2021;76:189–98. PubMed https://doi.org/10.1093/jac/dkaa393

3. Jones JC, Andreev K, Fabrizio TP, Bowman AS, Govorkova EA, Webby RJ. Baloxavir improves disease outcomes in mice after intranasal or ocular infection with influenza A virus H5N1-contaminated cow's milk. Nat Microbiol. 2025;10:836–40. PubMed https://doi.org/10.1038/s41564-025-01961-5

4. Andreev K, Jones JC, Kandeil A, Vogel P, Webby RJ, Govorkova EA. Baloxavir outperforms oseltamivir, favipiravir, and amantadine in treating lethal influenza A(H5N1) HA clade 2.3.4.4b infection in mice. Nat Commun. 2026;17:2937. PubMed https://doi.org/10.1038/s41467-026-69721-5

5. Bray NL, Pimentel H, Melsted P, Pachter L. Near-optimal probabilistic RNA-seq quantification. Nat Biotechnol. 2016;34:525–7. PubMed https://doi.org/10.1038/nbt.3519

6. Robinson MD, McCarthy DJ, Smyth GK. edgeR: a Bioconductor package for differential expression analysis of digital gene expression data. Bioinformatics. 2010;26:139–40. PubMed https://doi.org/10.1093/bioinformatics/btp616

7. Ihaka R, Gentleman RR. R: a language for data analysis and graphics. J Comput Graph Stat. 1996;5:299–314. https://doi.org/10.1080/10618600.1996.10474713

8. Sherman BT, Hao M, Qiu J, Jiao X, Baseler MW, Lane HC, et al. DAVID: a web server for functional enrichment analysis and functional annotation of gene lists (2021 update). Nucleic Acids Res. 2022;50(W1):W216–21. PubMed https://doi.org/10.1093/nar/gkac194

Appendix Table 1. Statistical parameters for AUC of weight change in study of A/mink/Spain/3691–8_22VIR10586–10/2022–infected mice*

Group (n = 7)	p-value	95% CI of diff
Vehicle vs		
OSP, 25 mg/kg	0.07	−729.5 to 27.54
OSP, 50 mg/kg	0.004	−1057 to −291.5
MPV, 25 mg/kg	0.03	−839.4 to −46.84
MPV, 50 mg/kg	0.005	−1221 to −325.7
BXA, 20 mg/kg	0.003	−1174 to −351.0
BXA, 50 mg/kg	0.009	−1206 to −253.4
OSP+MPV, 25+25 mg/kg	<0.001	−1172 to −462.4
OSP+MPV, 50+50 mg/kg	<0.001	−1287 to −563.2
BXA+OSP, 20+25 mg/kg	<0.001	−1190 to −949.5
BXA+OSP, 20+50 mg/kg	<0.001	−1193 to −941.7
BXA+MPV, 20+25 mg/kg	<0.001	−1174 to −933.2
BXA+MPV, 20+50 mg/kg	<0.001	−1190 to −949.5

*Abbreviations: AUC, area under the curve; BXA, baloxavir acid; CI, confidence interval; OSP, oseltamivir phosphate; MPV, molnupiravir

Appendix Table 2. Statistical parameters for Kaplan-Meier survival curves in study of A/mink/Spain/3691–8_22VIR10586–10/2022–infected mice*

Group (n = 7)	MST, d	p-value	HR	95% CI of ratio
Vehicle vs	9.0			
OSP, 25 mg/kg	11.0	0.02	0.17	0.04 to 0.77
OSP, 50 mg/kg	≥18.0	0.001	0.07	0.01 to 0.35
MPV, 25 mg/kg	11.0	0.005	0.11	0.02 to 0.50
MPV, 50 mg/kg	≥18.0	0.003	0.09	0.02 to 0.45
BXA, 20 mg/kg	≥18.0	0.001	0.06	0.01 to 0.33
BXA, 50 mg/kg	≥18.0	0.01	0.14	0.03 to 0.63
OSP+MPV, 25+25 mg/kg	≥18.0	<0.001	0.05	0.01 to 0.29
OSP+MPV, 50+50 mg/kg	≥18.0	<0.001	0.06	0.01 to 0.33
BXA+OSP, 20+25 mg/kg	≥18.0	<0.001	0.04	0.01 to 0.21
BXA+OSP, 20+50 mg/kg	≥18.0	<0.001	0.04	0.01 to 0.21
BXA+MPV, 20+25 mg/kg	≥18.0	<0.001	0.04	0.01 to 0.21
BXA+MPV, 20+50 mg/kg	≥18.0	<0.001	0.04	0.01 to 0.21

*Abbreviations: BXA, baloxavir acid; CI, confidence interval; HR, hazard ratio; MPV, molnupiravir; MST, median survival time; OSP, oseltamivir phosphate

Appendix Table 3. Statistical parameters for lung viral titers at 4 and 6 dpi in study of A/mink/Spain/3691–8_22VIR10586–10/2022–infected mice*

	4 d post infection		6 d post infection	
Group (n = 7)	p-value	95% CI of diff	p-value	95% CI of diff
Vehicle vs				
OSP, 25 mg/kg	<0.001	1.01 to 3.66	0.97	−1.22 to 2.38
OSP, 50 mg/kg	<0.001	2.09 to 4.74	0.007	0.45 to 4.05
MPV, 25 mg/kg	0.16	−0.24 to 2.41	0.04	0.034 to 3.63
MPV, 50 mg/kg	0.007	0.34 to 2.99	0.001	0.78 to 4.38
BXA, 20 mg/kg	<0.001	1.84 to 4.49	0.25	−0.46 to 3.13
BXA, 50 mg/kg	<0.001	2.18 to 4.83	<0.001	1.12 to 4.72
OSP+MPV, 25+25 mg/kg	0.16	−0.24 to 2.41	0.15	−0.30 to 3.30
OSP+MPV, 50+50 mg/kg	0.05	0.008 to 2.66	0.02	0.20 to 3.80
BXA+OSP, 20+25 mg/kg	<0.001	2.76 to 5.41	<0.001	1.70 to 5.30
BXA+OSP, 20+50 mg/kg	<0.001	3.09 to 5.74	<0.001	2.28 to 5.88
BXA+MPV, 20+25 mg/kg	<0.001	1.93 to 4.58	0.01	0.37 to 3.97
BXA+MPV, 20+50 mg/kg	<0.001	2.68 to 5.33	<0.001	2.70 to 6.30

*Abbreviations: BXA, baloxavir acid; CI, confidence interval; MPV, molnupiravir; OSP, oseltamivir phosphate

Appendix Table 4. Statistical parameters for brain viral titers at 4 and 6 dpi in study of A/mink/Spain/3691–8_22VIR10586–10/2022–infected mice*

	4 d post infection		6 d post infection	
Group (n = 7)	p-value	95% CI of diff	p-value	95% CI of diff
Vehicle vs				
OSP, 25 mg/kg	0.82	−0.74 to 1.91	0.02	0.28 to 3.88
OSP, 50 mg/kg	0.82	−0.74 to 1.91	<0.001	1.87 to 5.47
MPV, 25 mg/kg	0.82	−0.74 to 1.91	<0.001	1.28 to 4.88
MPV, 50 mg/kg	0.82	−0.74 to 1.91	<0.001	1.62 to 5.22
BXA, 20 mg/kg	0.82	−0.74 to 1.91	<0.001	1.87 to 5.47
BXA, 50 mg/kg	0.82	−0.74 to 1.91	<0.001	1.87 to 5.47
OSP+MPV, 25+25 mg/kg	0.82	−0.74 to 1.91	<0.001	1.87 to 5.47
OSP+MPV, 50+50 mg/kg	0.82	−0.74 to 1.91	<0.001	1.87 to 5.47
BXA+OSP, 20+25 mg/kg	0.82	−0.74 to 1.91	<0.001	1.87 to 5.47
BXA+OSP, 20+50 mg/kg	0.82	−0.74 to 1.91	<0.001	1.87 to 5.47
BXA+MPV, 20+25 mg/kg	0.82	−0.74 to 1.91	<0.001	1.87 to 5.47
BXA+MPV, 20+50 mg/kg	0.82	−0.74 to 1.91	<0.001	1.87 to 5.47

*Abbreviations: BXA, baloxavir acid; CI, confidence interval; MPV, molnupiravir; OSP, oseltamivir phosphate

Appendix Table 5. Statistical parameters for heart viral titers at 4 and 6 dpi in study of A/mink/Spain/3691–8_22VIR10586–10/2022–infected mice*

	4 d post infection		6 d post infection	
Group (n = 7)	p-value	95% CI of diff	p-value	95% CI of diff
Vehicle vs				
OSP, 25 mg/kg	0.43	−0.49 to 2.16	<0.001	0.87 to 4.47
OSP, 50 mg/kg	0.43	−0.49 to 2.16	<0.001	1.12 to 4.72
MPV, 25 mg/kg	0.43	−0.49 to 2.16	<0.001	1.12 to 4.72
MPV, 50 mg/kg	0.43	−0.49 to 2.16	<0.001	1.12 to 4.72
BXA, 20 mg/kg	0.43	−0.49 to 2.16	<0.001	1.12 to 4.72
BXA, 50 mg/kg	0.43	−0.49 to 2.16	<0.001	1.12 to 4.72
OSP+MPV, 25+25 mg/kg	0.43	−0.49 to 2.16	<0.001	1.12 to 4.72
OSP+MPV, 50+50 mg/kg	0.43	−0.49 to 2.16	<0.001	1.12 to 4.72
BXA+OSP, 20+25 mg/kg	0.43	−0.49 to 2.16	<0.001	1.12 to 4.72
BXA+OSP, 20+50 mg/kg	0.43	−0.49 to 2.16	<0.001	1.12 to 4.72
BXA+MPV, 20+25 mg/kg	0.43	−0.49 to 2.16	<0.001	1.12 to 4.72
BXA+MPV, 20+50 mg/kg	0.43	−0.49 to 2.16	<0.001	1.12 to 4.72

*Abbreviations: BXA, baloxavir acid; CI, confidence interval; MPV, molnupiravir; OSP, oseltamivir phosphate

Appendix Table 6. Bliss-independence interaction estimates for 2-drug antiviral regimens using end-of-study survival (18 dpi) as the efficacy endpoint (n = 7/group) in study of baloxavir-based combinations and monotherapies in treating clade 2.3.4.4b H5N1 infection in mice*

Monotherapy				Combination				
A (mg/kg)	Survival (n/N)	B (mg/kg)	Survival (n/N)	Regimens (mg/kg)	Survival (n/N)	Eexp (Bliss)	ΔBliss	Bootstrap 95% CI
OSP 25	2/7	MPV 25	2/7	OSP 25 + MPV 25	5/7	0.49	0.224	−0.24 to +0.71
BXA 20	4/7	OSP 25	2/7	BXA 20 + OSP 25	7/7	0.694	0.306	+0.06 to +0.61
BXA 20	4/7	MPV 25	2/7	BXA 20 + MPV 25	7/7	0.694	0.306	+0.06 to +0.61
OSP 50	4/7	MPV 50	5/7	OSP 50 + MPV 50	6/7	0.878	−0.02	−0.37 to +0.31
BXA 20	4/7	OSP 50	4/7	BXA 20 + OSP 50	7/7	0.816	0.184	+0.00 to +0.49
BXA 20	4/7	MPV 50	5/7	BXA 20 + MPV 50	7/7	0.878	0.122	+0.00 to +0.37

*Abbreviations: BXA, baloxavir acid; CI, confidence interval; dpi, days post infection; MPV, molnupiravir; OSP, oseltamivir phosphate

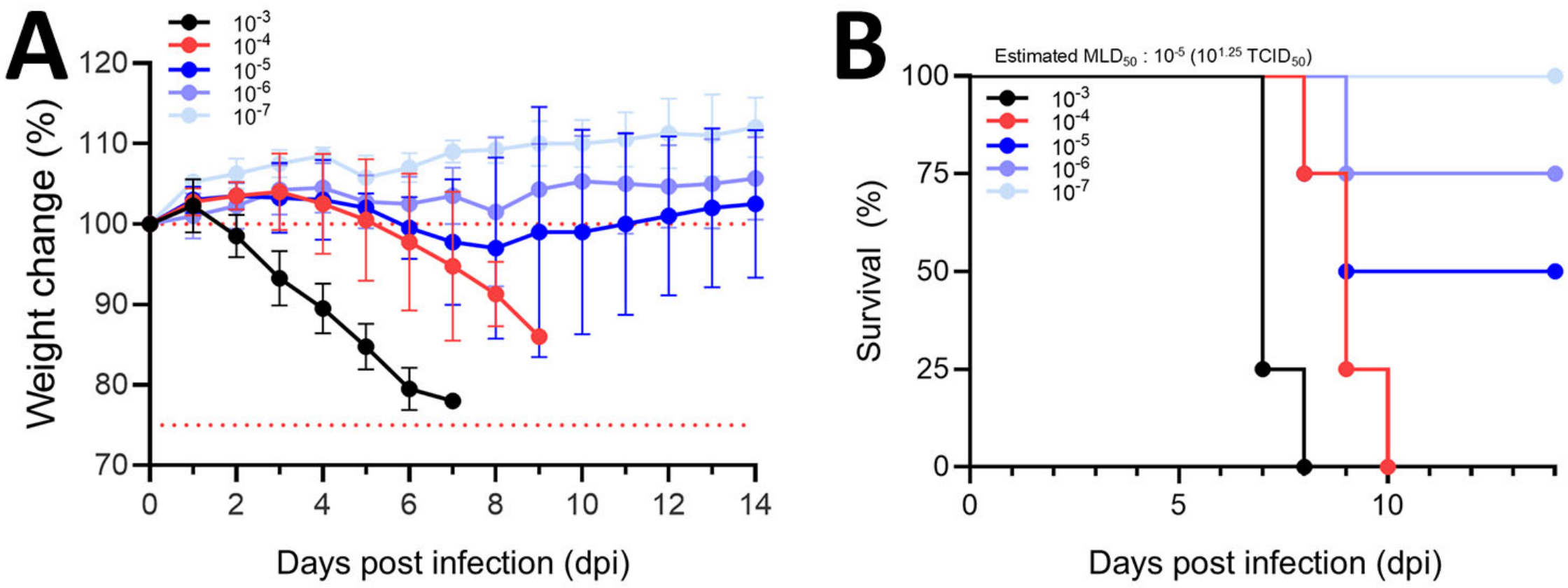

Appendix Figure 1. Determination of the MLD_{50} for clade 2.3.4.4b H5N1 challenge. Six- to 8-week-old female BALB/c mice were intranasally inoculated with 10-fold serial dilutions of virus (10^{-3} to 10^{-7}; n = 4/dilution) and monitored for bodyweight and survival. A) bodyweight change over time (mean±SD); red dotted line indicates the humane endpoint threshold. B) Kaplan–Meier survival curves. The estimated MLD_{50} is indicated on the survival panel and was used to define the 5 MLD_{50} challenge dose in the therapeutic experiments. Abbreviations: MLD, mouse lethal dose; SD, standard deviation.

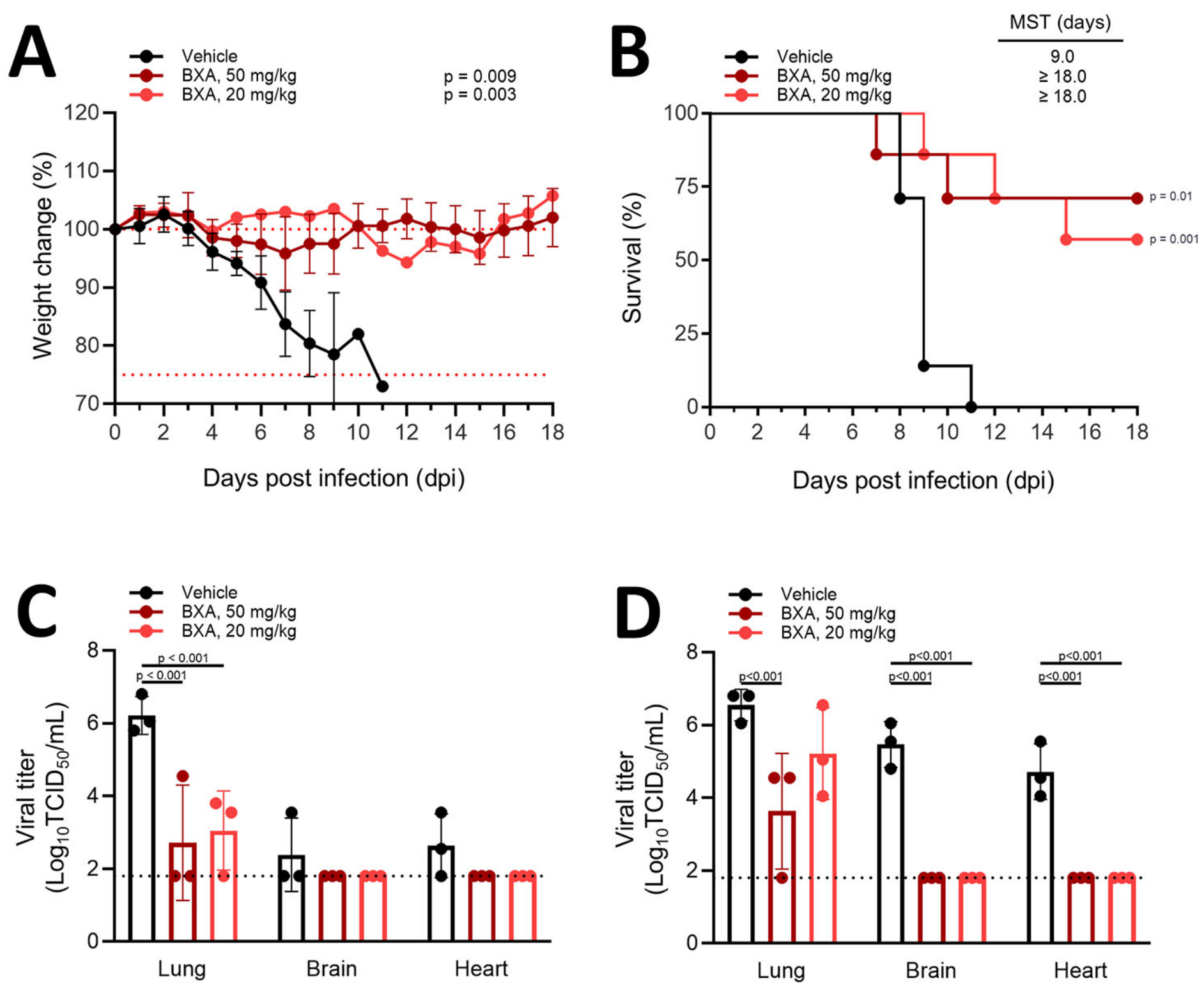

Appendix Figure 2. Comparison of baloxavir acid monotherapy at 20 and 50 mg/kg improves outcomes but does not fully prevent mortality. Mice were infected as in Figure 1 and treated at 6 hpi with a single subcutaneous dose of BXA 50 and 20 mg/kg; vehicle controls were processed in

parallel. A) Bodyweight change (mean±SD; n = 7/group) through 18 dpi; p value indicates AUC comparison versus vehicle. B) Kaplan–Meier survival (n = 7/group) with MST values shown. C) Viral titers in lung, brain, and heart at 4 dpi (n = 3/group). D) Viral titers at 6 dpi (n = 3/group). Viral titers were quantified by $TCID_{50}$ assay on MDCK cells; bars show group means with individual animals overlaid; dashed line indicates the assay limit of detection (1.801 $Log_{10}TCID_{50}$/mL). Statistics and significance notation are as described for Figure 1. Abbreviations: hpi, hours post infection; BXA, baloxavir acid; SD, standard deviation; AUC, area under the curve; dpi, days post infection; MST, median survival time; TCID, tissue culture infectious dose.

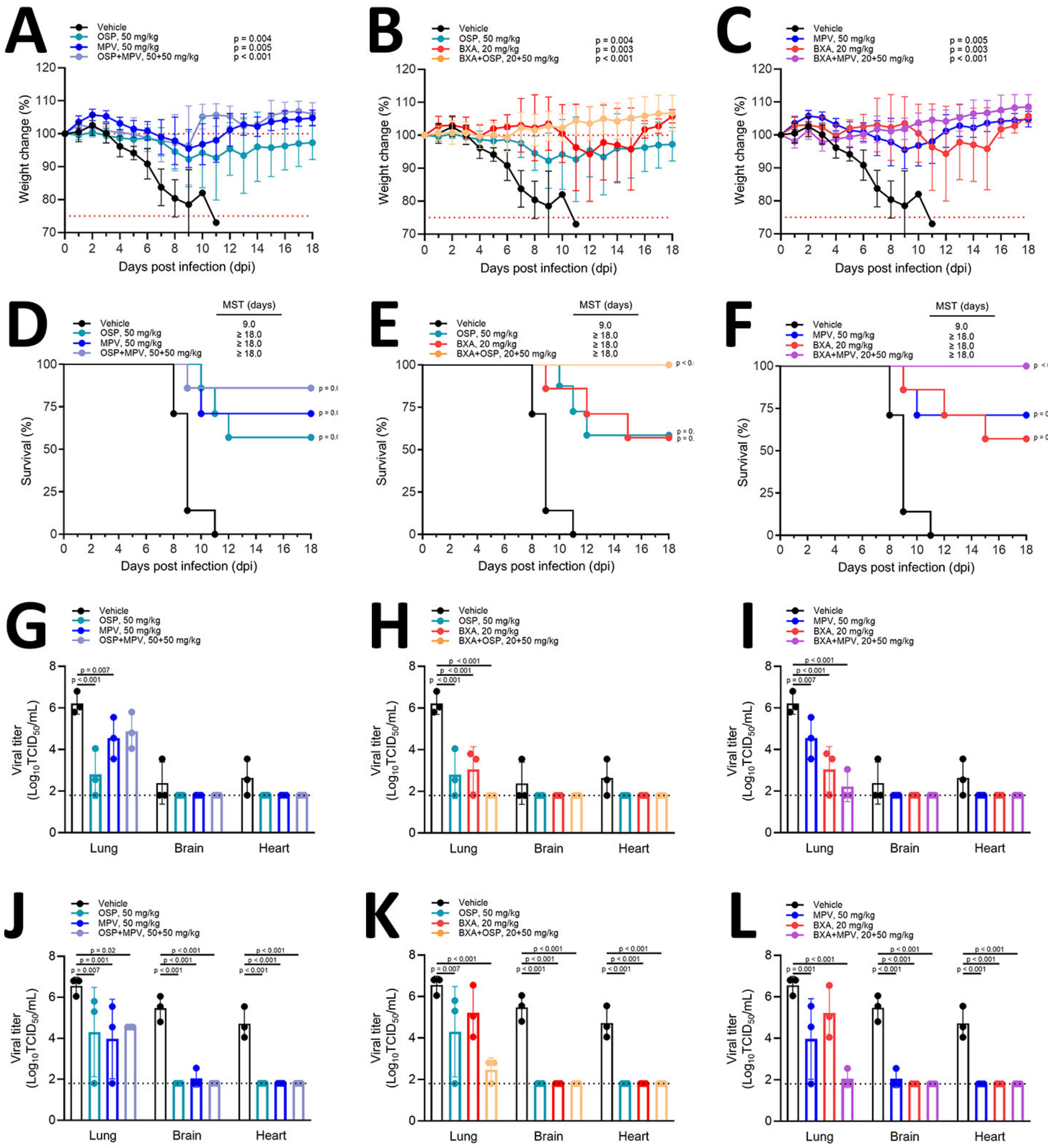

Appendix Figure 3. Therapeutic efficacy under a higher-dose regimen supports consistent benefit of BXA-containing combinations. Experimental design as in Figure 1, except for the dosing regimen: OSP and MPV were administered at 50 mg/kg (alone or as 50+50 mg/kg), while BXA was maintained at 20 mg/kg in BXA-containing combinations (20+50 mg/kg). BXA monotherapy in this regimen

remained 20 mg/kg (as in Figure 1); higher-dose BXA monotherapy is shown separately (Appendix Figure 2). Treatment started at 6 hpi (OSP/MPV oral twice daily for 5 days; BXA subcutaneous once). A–C) Bodyweight change (mean±SD; n = 7/group) through 18 dpi. (A) Vehicle, MPV 50, OSP 50, OSP+MPV 50+50 mg/kg. (B) Vehicle, OSP 50, BXA 20, BXA+OSP 20+50 mg/kg. (C) Vehicle, MPV 50, BXA 20, BXA+MPV 20+50 mg/kg. D–F) Kaplan–Meier survival (n = 7/group) corresponding to panels A–C; MST values are shown. G–I) Viral titers in lung, brain, and heart at 4 dpi (n = 3/group). J–L) Viral titers at 6 dpi (n = 3/group). Titers were quantified by $TCID_{50}$ assay; bars show group means with individual animals overlaid; dashed line indicates the assay limit of detection (1.801 $Log_{10}TCID_{50}$/mL). Statistics and significance notation are as described for Figure 1. Abbreviations: BXA, baloxavir acid; hpi, hours post infection; OSP, oseltamivir phosphate; MPV, molnupiravir; SD, standard deviation; dpi, days post infection; MST, median survival time; AUC, area under the curve; TCID, tissue culture infectious dose.
